# Stability of the H-cluster under whole-cell conditions—formation of an H_trans_-like state and its reactivity towards oxygen

**DOI:** 10.1007/s00775-022-01928-5

**Published:** 2022-03-08

**Authors:** Marco Lorenzi, Pierre Ceccaldi, Patricia Rodríguez-Maciá, Holly Jayne Redman, Afridi Zamader, James A. Birrell, Livia S. Mészáros, Gustav Berggren

**Affiliations:** 1 Molecular Biomimetics, Department of Chemistry–Ångström Laboratory, Uppsala University, Box 523, 75120 Uppsala, Sweden; 2 Present Address: Current Address: R&I Consultant, Home Office, Marseille, France; 3 Department of Inorganic Spectroscopy, Max Planck Institute for Chemical Energy Conversion, Stiftstrasse 34-36, 45470 Mülheim an der Ruhr, Germany; 4 Present Address: Current address: Department of Chemistry, Inorganic Chemistry Laboratory, University of Oxford, South Parks Road, Oxford, OX1 3QR UK; 5 Laboratoire de Chimie et Biologie des Métaux, Université Grenoble Alpes, CNRS, CEA, 17 rue des Martyrs, 38054 Grenoble, France

**Keywords:** Metalloenzymes, Hydrogenase, Enzyme mechanism, Electron paramagnetic resonance (EPR), Biophysics

## Abstract

Hydrogenases are metalloenzymes that catalyze the reversible oxidation of molecular hydrogen into protons and electrons. For this purpose, [FeFe]-hydrogenases utilize a hexanuclear iron cofactor, the H-cluster. This biologically unique cofactor provides the enzyme with outstanding catalytic activities, but it is also highly oxygen sensitive. Under in vitro conditions, oxygen stable forms of the H-cluster denoted H_trans_ and H_inact_ can be generated via treatment with sulfide under oxidizing conditions. Herein, we show that an H_trans_-like species forms spontaneously under intracellular conditions on a time scale of hours, concurrent with the cells ceasing H_2_ production. Addition of cysteine or sulfide during the maturation promotes the formation of this H-cluster state. Moreover, it is found that formation of the observed H_trans_-like species is influenced by both steric factors and proton transfer, underscoring the importance of outer coordination sphere effects on H-cluster reactivity.

**Graphical abstract:**

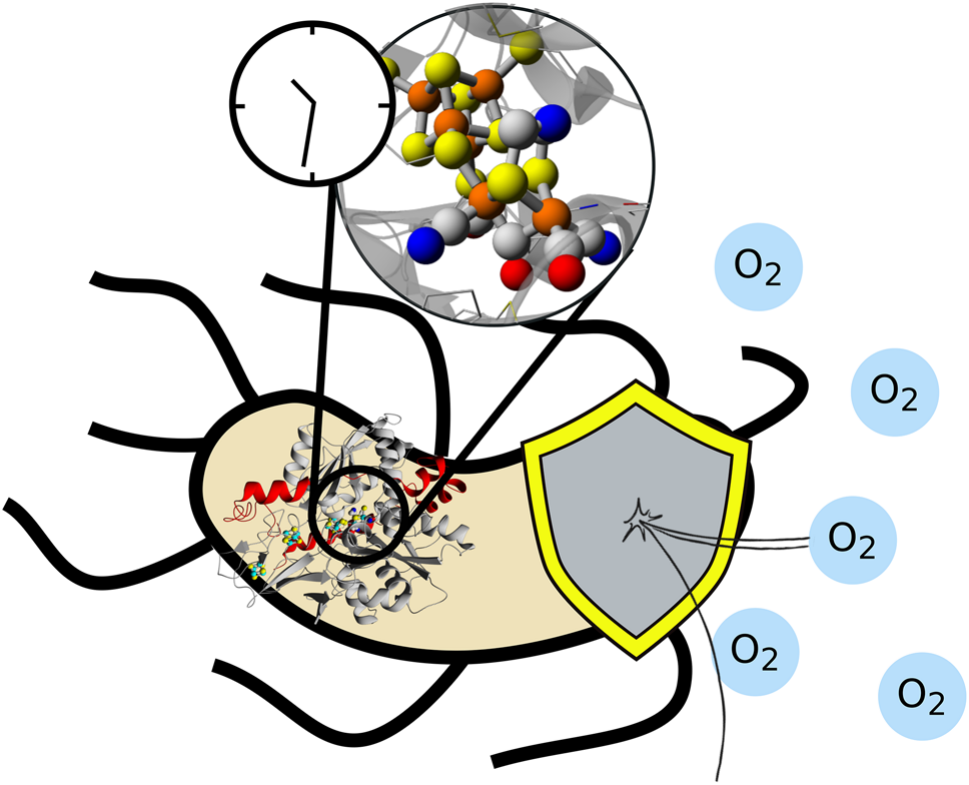

**Supplementary Information:**

The online version contains supplementary material available at 10.1007/s00775-022-01928-5.

## Introduction

**Fig. 1:**
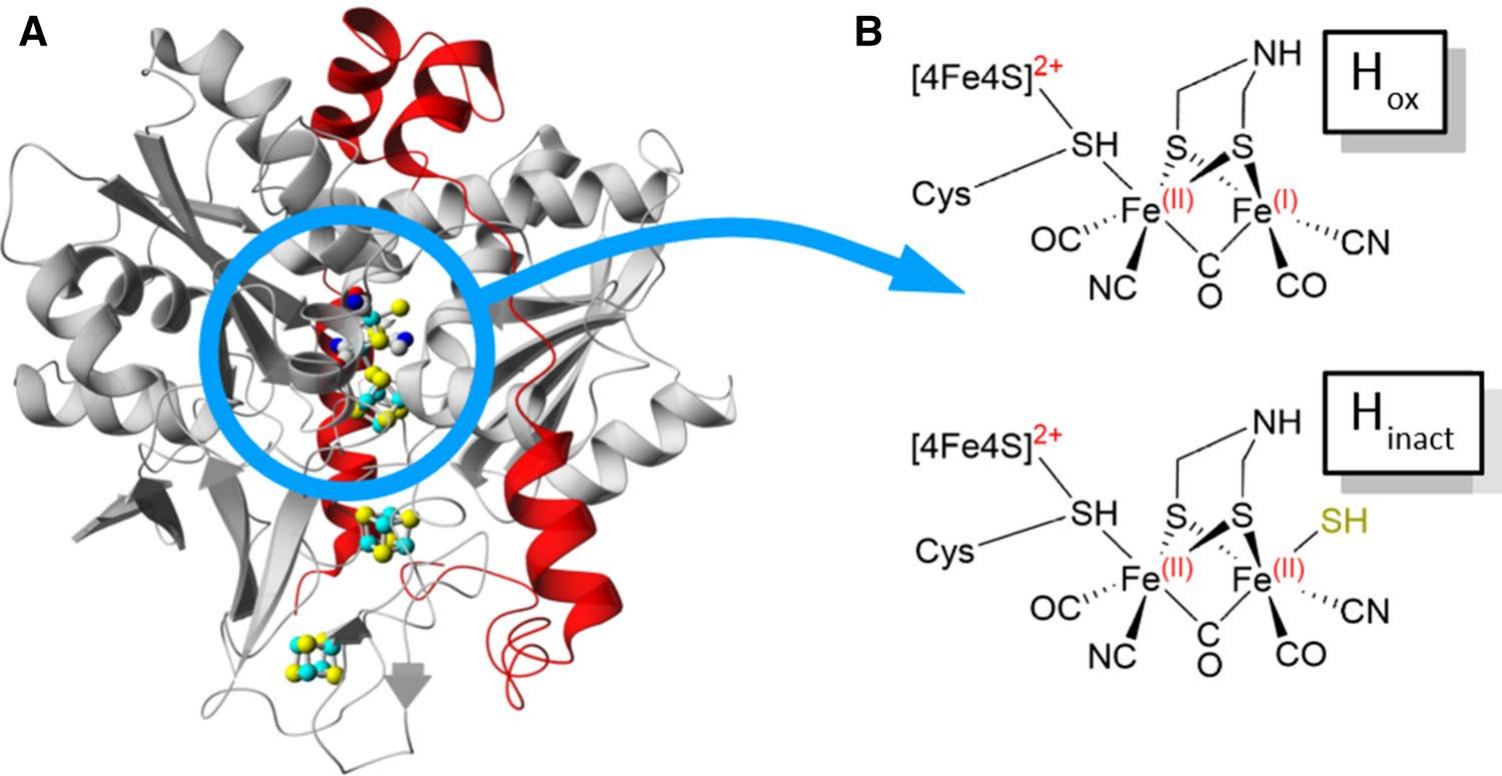
Schematic representation of [FeFe]-hydrogenase and the H-cluster. **A** The dimeric *Desulfovibrio desulfuricans* [FeFe]-hydrogenase (*Dd*HydAB) in the H_inact_ state, based on the crystal structure reported in ref [33] (PDB ascension number 6SG2). Subunit A is colored in gray, subunit B is colored in red, the color scheme for the H-cluster and for the accessory [FeS] clusters is based on the elements (Fe: cyan; S: yellow; N: blue; O: red; C: gray). **B** The H-cluster in the catalytically active state H_ox_ and in the inhibited state H_inact_

Hydrogen gas (H_2_) is a promising energy vector for the coming energy transition, due to its high energy/mass ratio and clean combustion [1, 2]. As of today, the lack of a cheap and efficient catalyst for H_2_ production prevents any possible large-scale application, since currently available technologies all rely on rare or precious elements [3–6]. [FeFe]-hydrogenases (Fig. 1A) are central to hydrogen metabolism in many microorganisms, as they catalyze both H_2_ oxidation and evolution [7]. Since their H_2_ evolution rates are as high as 10,000 s^−1^, these enzymes are also highly relevant in the context of developing sustainable biological and bio-inspired synthetic systems for H_2_/H^+^ interconversion [1, 2, 8–11]. The active site of [FeFe]-hydrogenases (the H-cluster, Fig. 1B) contains a unique diiron subcluster ([2Fe]_H_) where the low valent Fe ions are bridged by a bidentate azadithiolate ligand (^−^SCH_2_NHCH_2_S^−^, adt) and further coordinated by three CO and two CN^−^ ligands [2, 12–15]. The [2Fe]_H_ subsite is, in turn, attached to a canonical [4Fe-4S] cluster ([4Fe-4S]_H_) through a cysteine residue. The ligand geometry of the H-cluster leaves an open-coordination site on the [2Fe]_H_ subsite positioned in close proximity to the nitrogen of the adt ligand, and the latter is believed to act as a proton relay during catalysis. [FeFe]-hydrogenases are extremely O_2_ sensitive enzymes, and this sensitivity represents a key challenge for technological development. The precise inactivation mechanism is still under debate, but O_2_ is, with few exceptions, an irreversible inhibitor of the enzyme [16–21]. However, it is possible to protect the H-cluster by forming a reversibly inhibited state, as first observed already in the 1980s in the [FeFe]-hydrogenases from sulfate reducing bacteria including *Desulfovibrio vulgaris* and *Desulfovibrio desulfuricans* (*Dv*HydAB and *Dd*HydAB, respectively, whose protein sequences are identical), following isolation of the enzymes under aerobic conditions [22–25]. The chemical nature of this O_2_ stable form has since then been characterized in-depth under in vitro conditions [20, 26–30]. The catalytically active, but O_2_ sensitive, H_ox_ state has been observed in all [FeFe]-hydrogenases studied so far, and it exhibits an [Fe(II)Fe(I)]_H_ subsite while the [4Fe-4S]_H_-cluster resides in the oxidized (2 +) state [2, 9, 13, 27] (Fig. 1B). The H_ox_ state is also well-known for its affinity for CO, generating the reversibly inhibited H_ox_-CO state [31, 32]. In *Dd*HydAB the coordination of a sulfide ligand (SH^−^) to the [2Fe]_H_ subsite converts H_ox_ into the isoelectronic H_trans_ state ([4Fe-4S]_H_^+^-[Fe(II)Fe(II)-SH]_H_), which instead of degrading upon O_2_ exposure is oxidized to the so-called H_inact_ state ([4Fe-4S]_H_^2+^-[Fe(II)Fe(II)-SH]_H_), also referred to as H_ox_^air^ [33, 34] (Fig. 1B). Albeit an inhibited state, the H_inact_ state is readily reactivated following release of the sulfide ligand under reducing conditions [19, 27, 29, 35].

Despite having been widely studied in purified form, our insight into the catalytic cycle and stability of [FeFe]-hydrogenases in a cellular environment remains limited. Improved understanding of [FeFe]-hydrogenase reactivity under whole-cell conditions is critical not only to verify the physiological relevance of states observed in vitro, but also to improve the performance of hydrogenase-based biological and biohybrid energy applications. Herein, we take advantage of our capacity to generate semi-synthetic [FeFe]-hydrogenases in *E. coli* at concentrations suitable for whole-cell spectroscopy [9, 36–38], to investigate the formation of the H_trans_ and H_inact_ states under intracellular conditions. More specifically, we explore two different model enzymes: HydA1 from the green algae *Chlamydomonas reinhardtii* (*Cr*HydA1) and the aforementioned bacterial *Dd*HydAB. Moreover, a recent mechanistic proposal underscores the need for efficient proton-transfer during the formation of these sulfide-inhibited states [34]. Thus, we probe the influence of sterics and the proton-transfer network on the inhibition chemistry of the *Cr*HydA1 enzyme, through modifications of the organometallic cofactor as well as single-point mutations in the active-site pocket.

## Results and discussion

### Formation of an H_trans_-like state under whole-cell conditions

**Fig. 2:**
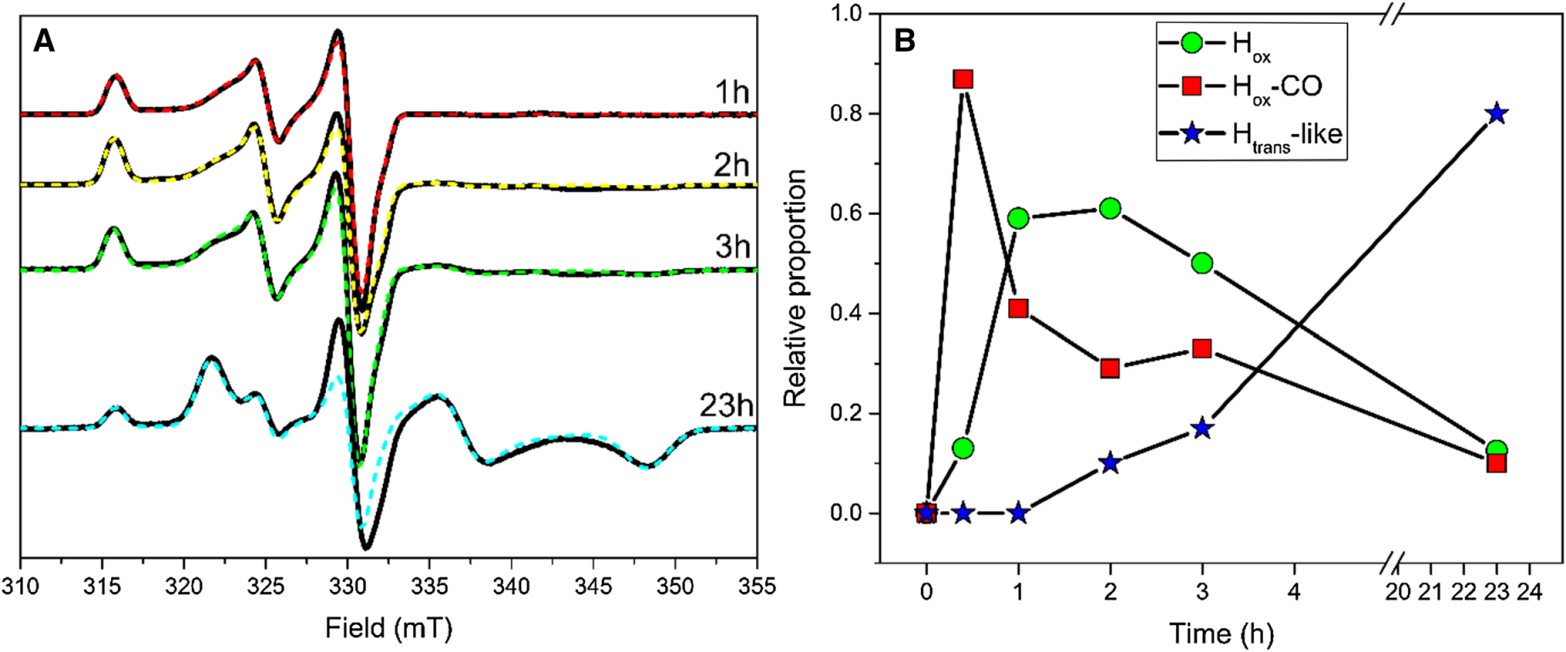
The effect of time on the population of H-cluster states in [2Fe]^adt^-*Cr*HydA1 monitored by EPR spectroscopy. **A** Whole-cell EPR spectra of [2Fe]^adt^-*Cr*HydA1 recorded on samples of *E. coli* cells expressing *apo-Cr*HydA1 and flash frozen following incubation with the [2Fe]^adt^ cofactor for 1 h, 2 h, 3 h and 23 h at 37 ˚C under anaerobic conditions. Experimental spectra from a representative dataset are overlaid with simulations (dashed lines), including H_ox_, H_ox_-CO and H_trans_ contributions. All spectra were corrected by subtracting the *apo-Cr*HydA1 control sample, to eliminate any signal coming from the cells and from unmatured *Cr*HydA1 (Fig. S4). The poor fitting around 330 mT on the 23 h spectrum is due to the imperfect subtraction of the variable *g* = 2.02 isotropic signal arising from the cell background, which is a minor contribution in the difference spectrum (see Fig. S4). EPR experimental conditions: T = 20 K, P = 1 mW, ν = 9.28 GHz. **B** Contribution of the different catalytic states to the signal intensity at the different time points on the same dataset. The relative contributions are calculated based on their respective weight in the simulations

To generate enzyme concentrations enabling H-cluster detection by electron paramagnetic resonance (EPR) spectroscopy *Cr*HydA1 was heterologously expressed in standard *E.coli* BL21, as previously described [36–39]. As *E. coli* does not natively express an [FeFe]-hydrogenase, the organism lacks the [FeFe]-hydrogenase-specific accessory proteins (HydEFG) and produces the enzyme in an inactive form, complete with the [4Fe-4S]_H_ cluster but lacking the diiron [2Fe]_H_ cofactor (*apo*-*Cr*HydA1) [40–42]. After protein overproduction, cell cultures were concentrated and the active semi-synthetic enzyme (holo-*Cr*HydA1, herein denoted [2Fe]^adt^-*Cr*HydA1) was subsequently generated through the addition of the synthetic [2Fe]_H_ cofactor mimic [Fe_2_(adt)(CO)_4_(CN)_2_]^2−^ ([2Fe]^adt^) under strictly anaerobic conditions. The final concentration of [2Fe]^adt^ in the cell suspension was 80 μM, as this has previously been shown to afford close to quantitative maturation of apo-*Cr*HydA1 under these conditions [36–39, 43]. The cell suspensions produced H_2_ for 1–2 h following the addition of [2Fe]^adt^, before slowing down significantly and halting production after 3–4 h (Fig. S1). In parallel to quantifying H_2_ production, whole-cell samples for EPR analysis were collected and flash frozen at different time points to monitor the formation and stability of the [2Fe]^adt^-*Cr*HydA1 holoenzyme over the course of 23 h. EPR spectra recorded on samples obtained after a 20 min incubation with [2Fe]^adt^ under an argon atmosphere displayed a mixture of one rhombic (g_z_ ≠ g_y_ ≠ g_x_) and one pseudo-axial (g_x_ = g_y_ ≠ g_z_) feature, respectively attributable to the H_ox_ (g_zyx_ = 2.100, 2.040, 1.998) and to the CO-inhibited H_ox_-CO states (g_∥**⊥**_ = 2.054, 2.007) [31] (Fig. 2). The H_ox_ and H_ox_-CO species continued to be the main constituents of the signal over the first 3 h, with the latter species gradually releasing CO and converting into the H_ox_ state. (Fig. 2B).

However, after 23 h of incubation, a significant decrease in the features specific to these two states is observed, concomitantly with the rise of a relatively broad rhombic signal with g_zyx_ = 2.064, 1.972, 1.910. Simulations show that the latter species begins to form already after 2 h of incubation, at which point it represents ~ 10% of the total spin count. This rhombic signal accumulates with time to then become the dominant species in the sample, contributing for ~ 80% of the total signal intensity after 23 h. At this point, the cells are able to resume H_2_ production when re-suspended in fresh media containing glucose (Fig. S1); however, the media exchange has no effect on the relative proportion of the different states (Fig. S2). Similarly, the broad rhombic signal displayed negligible changes when parallel samples incubated for 23 h were exposed to strongly reducing conditions via the addition of sodium dithionite and 1 atm H_2_ (data not shown). In combination, these observations suggest that, once formed, the species giving rise to the rhombic signal is highly stable. Thus, the H_2_ produced by aged cells is attributed to the small residual population still residing in the catalytically competent H_ox_ state.

The broad rhombic EPR signal (g_zyx_ = 2.064, 1.972, 1.910) was not observed when cells expressing apo-*Cr*HydA1 were incubated for up to 23 h in the absence of [2Fe]^adt^, nor in standard BL21 cells incubated with the same complex (Fig. S3), supporting the assignment of the signal to an H-cluster species. The g values of this new broad EPR signal are in good agreement with a signal previously attributed to the sulfide-inhibited H_trans_ state in *Dd*HydAB (g_zyx_ = 2.060; 1.960; 1.890) [25, 29]. To assess whether the origin of this new signal could be attributed to the binding of a sulfide or hydrosulfide ligand to the diiron subcluster, 375 μM Na_2_S were added to the incubation mixture concomitantly with [2Fe]^adt^. The addition of sulfide increased the overall intensity of the rhombic H_trans_-like signal, as reported for the in vitro formation of the H_trans_ state in *Dd*HydAB [34]. The same effect could be observed using L-cysteine as the source for the sulfide, albeit with a lower magnitude (Fig. S5).

### Intracellular H_trans_ formation in ***Dd***HydAB

**Fig. 3:**
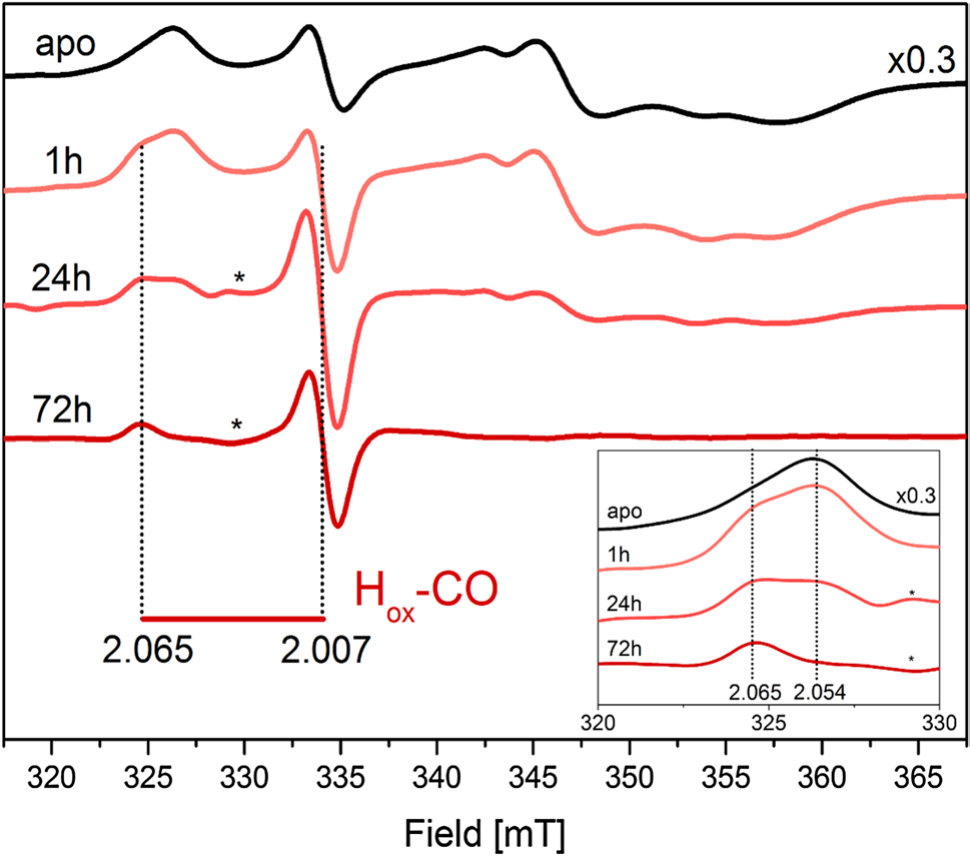
The effect of time on the population of H-cluster states in [2Fe]^adt^-*Dd*HydAB monitored by EPR spectroscopy. Whole-cell EPR spectra of apo- and [2Fe]^adt^-*Dd*HydAB recorded on samples of *E. coli* cells expressing apo-*Dd*HydAB and flash frozen prior to or following incubation with the [2Fe]^adt^ cofactor for 1 h, 24 h and 72 h under anaerobic conditions at 37 °C. The figure reports the g values for the H_ox_-CO state. (Inset): zoom in on the 320–330 mT area. Vertical dotted lines indicate the g = 2.065 feature belonging to H_ox_-CO and the g = 2.054 from the [4Fe-4S] clusters interaction spectrum. Asterisks mark a feature attributable to the (F_red_)H_ox_ interaction spectrum. EPR experimental conditions: T = 20 K, P = 1 mW, ν = 9.28 GHz. Note that the spectrum of apo-*Dd*HydAB has been normalized by a factor 0.3

The heterodimeric *Dd*HydAB hydrogenase has been the primary model system for elucidating the chemistry of the H_trans_ and H_inact_ states [29, 33, 34]. Thus, this enzyme was also studied under intracellular conditions for comparative purposes, with *Dd*HydAB overproduced in *E. coli* using the same methods as for *Cr*HydA1. In addition to the [4Fe-4S]_H_ component of the H-cluster, *Dd*HydAB features two additional [4Fe-4S] “F-clusters” serving as electron relays from the protein surface to the active site (Fig. 1A). The apo-*Dd*HydAB protein, containing only the three [4Fe-4S] clusters, shows an interaction spectrum of the [4Fe-4S] clusters’ signals under intracellular conditions (Fig. 3, apo-*Dd*HAB), as observed earlier for the purified enzyme [44]. In vitro activation of *apo*-*Dd*HydAB using [2Fe]^adt^ to generate [2Fe]^adt^-*Dd*HydAB (i.e. *holo*-*Dd*HydAB) is a relatively slow process. The mechanistic rationale for this remains to be elucidated, but is arguably attributable to the relative stabilities of the so-called “open” and “closed” conformations of the enzyme [44]. Nevertheless, treating apo-*Dd*HydAB expressing *E. coli* cells with [2Fe]^adt^ for just 1 h yielded a complex spectrum, with an axial signature attributable to an H_ox_-CO species clearly discernible under the dominant [4Fe-4S] interaction spectrum. The reason for the apparent higher rate of H-cluster assembly in *Dd*HydAB under whole-cell conditions is unknown. Extending the incubation time to 24 h resulted in a decrease of the [4Fe-4S] signals, possibly due to gradual oxidation, allowing also for the detection of a contribution arising from a small population of [2Fe]^adt^-*Dd*HydAB featuring the H-cluster in the H_ox_ state and a one-electron-reduced F-cluster (denoted (F_red_)H_ox_) [45]. However, no features attributable to H_trans_ or an H_trans_-like state were observed, even when extending the incubation time to 72 h, at which point the only remaining H-cluster signal is H_ox_-CO (Fig. 3). These experiments verify that [2Fe]^adt^-*Dd*HydAB can be generated through artificial maturation under whole-cell conditions, at a seemingly faster rate than during normal in vitro studies. Still, the lack of any H_trans_-like species in the case of *Dd*HydAB shows that the stability of this state under intracellular conditions can vary significantly between different [FeFe]-hydrogenases, as expected from variations in structure as well as reported affinities for other inhibitors such as carbon monoxide [46].

### Reactivity of the H_trans_-like state towards molecular oxygen.

**Fig. 4:**
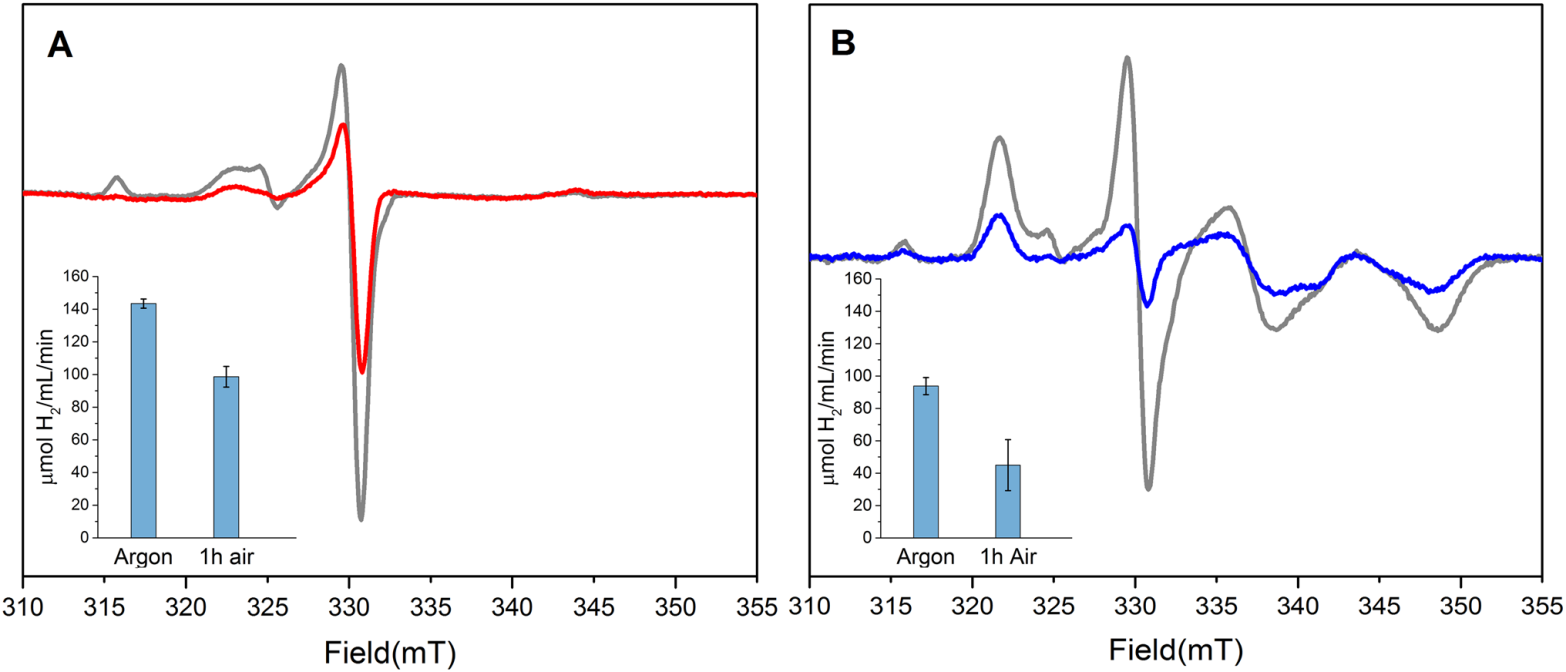
Reactivity towards molecular oxygen of whole-cell [2Fe]^adt^-*Cr*HydA1 samples. **Panel A:** The EPR spectrum of a whole-cell [2Fe]^adt^-*Cr*HydA1 sample initially incubated for 1 h with [2Fe]^adt^ under anaerobic conditions, and subsequently exposed to air for 1 h **(red spectrum)** is overlaid with an equivalent sample kept strictly anaerobic **(gray spectrum).** (**Inset**): Enzymatic activities for the same samples measured through in vitro assays. **Panel B:** The EPR spectrum of a whole-cell [2Fe]^adt^-*Cr*HydA1 sample initially incubated for 23 h with [2Fe]^adt^ under anaerobic conditions, and subsequently exposed to air for 1 h at ambient temperature **(blue spectrum)** is overlaid with an equivalent sample kept anaerobic **(gray spectrum).** (**Inset**): Enzymatic activities for the same samples measured through in vitro assays. EPR data shown come from representative spectra. Activity data represent the average values of two biological replicates with standard deviations reported as error bars. EPR experimental conditions: T = 20 K, P = 1 mW, ν = 9.28 GHz

As mentioned in the introduction, in *Dd*HydAB the H_inact_ state forms via H_trans_ under oxidative conditions (e.g. during aerobic purification) and has been demonstrated to grant protection from O_2_-induced H-cluster degradation. Conversely, *Cr*HydA1 is known to be an extremely oxygen-sensitive enzyme and aerobic purification of an active enzyme has not been reported [20]. Still, *Cr*HydA1 has also been shown to be able to enter the O_2_-protected H_inact_ state under controlled in vitro conditions [34]. To examine if exposing whole-cell [2Fe]^adt^-*Cr*HydA1 samples to molecular oxygen could trigger the formation of H_inact_ or H_trans_, and whether these states were able to grant resistance towards O_2_ damage, cell suspensions were exposed to air and the effects on the H-cluster were monitored via EPR spectroscopy and enzyme activity assays. As described above, incubating cells expressing apo-*Cr*HydA1 with [2Fe]^adt^ for 1 h under anaerobic conditions, generates an enzyme population consisting of H_ox_ and H_ox_-CO. Subsequent exposure of such samples to O_2_, by placing them in air for 1 h, resulted in a noticeable decrease of the overall EPR signal intensity (total residual signal intensity ≈ 45%) (Fig. 4, panel A). Visibly, all of the residual signal is attributable to the H_ox_-CO state; this is expected as it is known to be more resistant to oxygen and to be formed by CO released upon H-cluster degradation (a process known as “cannibalization”) [27, 29]. The same air exposure treatment on samples pre-incubated under argon for 23 h, in which the H-cluster resides primarily in the H_trans_-like state, yielded a similar result, with a decrease in the intensity of the spectral features of both the H_trans_-like species and residual H_ox_ (total residual signal intensity ≈ 35%) (Fig. 4, panel B). This loss of EPR signal intensity can be attributed either to the formation of an EPR silent H_inact_-like state or to O_2_-induced degradation of the H-cluster. To separate these two possibilities, the EPR experiments were complemented with in vitro activity assays on lysed cells. The use of a strong reductant (dithionite), in combination with methyl viologen as electron mediator, is expected to re-activate any H-cluster fraction residing in H_inact_ or H_trans_, and thus enable quantification of the entire intact H-cluster population.

It should be noted that already prior to O_2_ exposure the 23 h pre-incubated samples did display slightly diminished activities, as compared to the 1 h pre-incubated samples (compare Fig. 4A and B, insets). We attribute this to H-cluster degradation, as in vitro assays show no indication of degradation processes involving the protein scaffold on a 24 h time scale (Fig. S4). The readily observable H_2_ production from the lysed cells enable a relative quantification of intact enzyme to determine the effect of O_2_ on the different preparations. Regardless of pre-incubation time (1 or 23 h), the signal loss observed by EPR spectroscopy following oxygen exposure was larger than the activity loss. The 1 h-pre-incubated sample retained ~ 70% of the H_2_-producing activity and the 23 h-pre-incubated sample retained ~ 50% of its activity, when compared to parallel samples kept under strictly anaerobic conditions (Fig. 4, insets).

Consequently, the observed EPR signal loss is attributable to a combination of H-cluster degradation and formation of an EPR silent H_inact_-like state. Considering the well-established reactivation of H_inact_ under reducing conditions [29, 34], we expect the latter species to re-enter the catalytic cycle under the conditions employed in the in vitro activity assays, while the former causes a definitive loss in activity. Moreover, pre-established accumulation of the H_trans_-like state did not provide any apparent increase in O_2_-tolerance, as samples pre-incubated for 23 h showed larger relative activity losses as compared to samples pre-incubated only for 1 h. In contrast, the cellular environment showed remarkable capabilities in terms of O_2_-protection, as a significant fraction of the H-cluster population was not degraded nor oxidized to an H_inact_-like state during oxygen exposure. This is attributed to the low intracellular O_2_ concentrations ensured by cellular respiration, a process which is indeed expected to be more efficient in “young” and metabolically active cells [47].

### Structural factors influencing formation of the H_trans_ and H_inact_-like states

The presence of an efficient proton-transfer chain has been proposed to be important for the formation of the H_trans_ and H_inact_ states, as the sulfide ligand is thought to enter the active-site pocket as H_2_S and to undergo a protein-assisted deprotonation event to yield the SH^−^ ligand [34]. Thus, modifications were made to both the [2Fe]_H_ subsite as well as to the active-site pocket to assess the importance of the proton-transfer pathway on formation of the H_trans_ and H_inact_-like states. More specifically, holo-*Cr*HydA1 samples were prepared in which the [2Fe]^adt^ mimic was replaced with [Fe_2_(pdt)(CO)_4_(CN)_2_]^2−^ ([2Fe]^pdt^, pdt = ^−^SCH_2_CH_2_CH_2_S^−^, propanedithiolate), generating [2Fe]^pdt^–*Cr*HydA1. In this modified H-cluster, the amine bridgehead present in the natural [2Fe]^adt^ cofactor is substituted with a non-protonatable methylene group. Earlier whole-cell and in vitro spectroscopic studies, as well as crystallographic studies, have shown that [2Fe]^pdt^–*Cr*HydA1 is an organometallic variant where the [2Fe] subsite is locked in an oxidized Fe(II)Fe(I) state, strikingly similar to the EPR active H_ox_ state of the native enzyme [14, 37–39, 43, 48, 49]. As expected, EPR samples consisting of apo-*Cr*HydA1-expressing cells incubated with the [2Fe]^pdt^ complex revealed the exclusive formation an H_ox_-like species. The H-cluster population remained locked in the H_ox_ state for up to 23 h of incubation under anaerobic conditions; and no features attributable to an H_trans_-like species were discernable at any point during the experiment (Fig. S6).

**Fig. 5:**
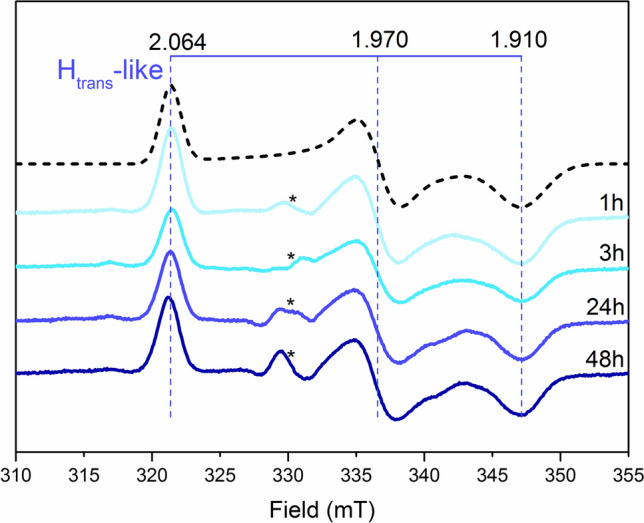
Monitoring the formation of the H_trans_-like state in [2Fe]^adt^-*Cr*HydA1-C169S under whole-cell conditions by EPR spectroscopy. EPR spectra recorded for *E. coli* cells expressing apo-*Cr*HydA1-C169S flash frozen after incubation with [2Fe]^adt^ for 1, 3, 24 or 48 h at 37 °C. A characteristic H_trans_-like signature became visible already after 1 h of incubation; a spectral simulation for this species is shown as a stacked dashed line and g values obtained from the fittings are reported. Unassigned, sample-specific weak signals potentially arising from the imperfect subtraction of the cell background are indicated with asterisks. EPR experimental conditions: T = 20 K, P = 1 mW, ν = 9.28 GHz

These results seem to confirm the importance of a proton relay in the second coordination sphere of the H-cluster for the formation of the H_trans_ and H_inact_-like states. To proceed one step further down the proton-transfer pathway, the C169 residue was mutated to a serine (*Cr*HydA1-C169S). This cysteine residue is strictly conserved in “prototypical” [FeFe]-hydrogenases, and it is the first amino acid involved in the proton-transfer chain towards the adt-nitrogen of the H-cluster [9]. The C169S mutant has been studied extensively under in vitro conditions, and demonstrated to be a variant with a severely compromised proton-transfer pathway and significantly decreased catalytic activity [50, 51]. Incubating *E. coli* cells expressing *Cr*HydA1-C169S with [2Fe]^adt^ resulted in the rapid formation of a broad rhombic EPR signal with g_zyx_ = 2.064; 1.970; 1.910, very closely matching the H_trans_-like signal in the wild-type enzyme (g_zyx_ = 2.064; 1.972; 1.910). A signal with comparable EPR properties has been reported from in vitro studies of the C169S mutant (g_zyx_ = 2.065; 1.969; 1.906) [51, 52]. It was originally assigned to an H_trans_-like species, potentially featuring a hydride ligand. However, despite the similarity in g values the signal observed under our in vivo conditions is highly unlikely to represent a metal hydride species. No other H-cluster derived features were discernable in the EPR spectrum (Fig. 5).

The evolution of this H_trans_-like signal over time was examined with long-term (1 h, 3 h, 23 h, 48 h) incubation of the cells with the [2Fe]^adt^ cofactor, and no significant change was detected neither in the shape, nor intensity of the signal (Fig. 5). The stability of this state was further confirmed by its isolation. In contrast to the wild-type protein, [2Fe]^adt^-*Cr*HydA1-C169S could be purified in its H_trans_-like state via affinity chromatography following anaerobic cell lysis. However, this required supplementing the buffers with 100 mM Na_2_S, as omitting sulfide resulted in a complete loss of the H_trans_-like rhombic EPR signal (Fig. S7). Albeit EPR spectroscopy revealed rapid H-cluster formation, no in vivo or in vitro hydrogen production could be detected from [2Fe]^adt^-*Cr*HydA1-C169S samples, as expected from this reportedly inactive variant [50].

To gain more insight into potential intermediates formed on route to the H_trans_-like state in the C169S mutant, the process was studied at lower temperature. More specifically, apo-*Cr*HydA1-C169S containing cells were treated with [2Fe]^adt^ at 12 ˚C. The first discernable state generated under these conditions was H_ox_ (20–60 min), while the H_trans_-like state only started to be detectable after 2 h of incubation with [2Fe]^adt^ (Fig. S8). The H_trans_-like signal reached full intensity after overnight incubation with the cofactor.

**Fig. 6:**
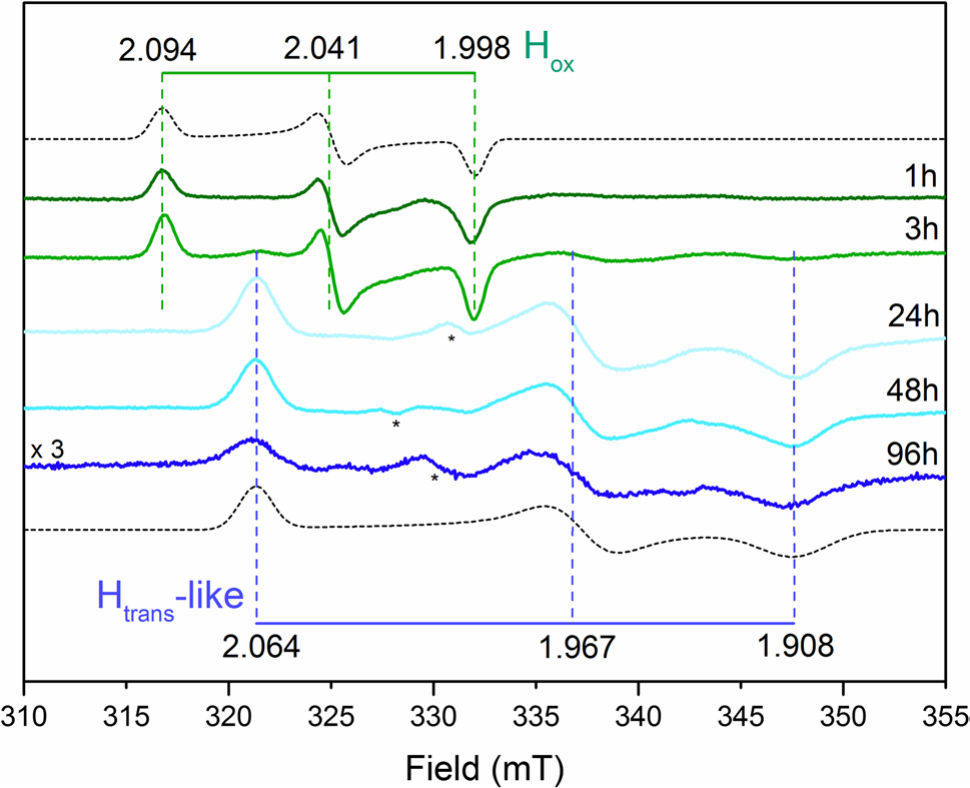
Monitoring the formation of the H_trans_-like state in *Cr*HydA1-C169S with the [2Fe]^pdt^ cofactor. EPR spectra of *E. coli* cells expressing apo-*Cr*HydA1-C169S after incubation with [2Fe]^pdt^ for variable amounts of time at 37 °C. The H_trans_-like signature only became visible after 23 h of incubation. Unassigned, sample-specific weak signals potentially arising from the imperfect subtraction of the cell background are indicated with asterisks. EPR experimental conditions: T = 20 K, P = 1 mW, ν = 9.28 GHz

As modifications of the bridgehead atom showed clearly diverging effects as compared to the C169S mutant, with regards to formation of the H_trans_-like state, the combined effect of the latter mutation and the methylene-bridgehead mimic was investigated. Whole-cell samples of the C169S mutant matured with [2Fe]^pdt^ ([2Fe]^pdt^-*Cr*HydA1-C169S) initially displayed a behavior consistent with the [2Fe]^pdt^ wild-type, with an EPR spectrum showing an H-cluster apparently locked in an H_ox_- like state (Fig. 6). However, a signal corresponding to an H_trans_-like state started to accumulate after 3 h of incubation and became predominant after 24 h. No other signals arose thereafter, on a time scale of days (96 h).

In summary, the [2Fe]^pdt^-variant of the wild-type *Cr*HydA1 does not form detectable amounts of any H_trans_ or H_inact_-like states, in agreement with previous studies performed in vitro [34]. Conversely, the [2Fe]^pdt^-*Cr*HydA1-C169S ‘double’ variant showed accumulation of the H_trans_-like state. This shows that the presence of a nitrogen-bridgehead facilitates, but is not critical, for the formation of these inhibited states. Rather, it underscores that additional factors in the outer coordination sphere also affect their formation. These include a decrease of steric bulk resulting from the replacement of a thiol group with an alcohol that provides easier access for exogenous ligands and a change in the hydrogen-bonding dynamics involving the first and second coordination spheres.

## Conclusions

Semi-synthetic [FeFe]-hydrogenases have been shown to remain catalytically active in cyanobacteria on a time scale of days [43, 53]. The observation that *E. coli* cells expressing *C. reinhardtii* [FeFe]-hydrogenase can produce hydrogen gas only transiently following enzyme activation has raised the question on the stability of these enzymes in the bacterial cytoplasm.

In the case of *Cr*HydA1, EPR spectra recorded on long time-scale samples show the gradual accumulation of an H_trans_-like state under the slow-turnover conditions assayed here. However, the rate of formation of this H_trans_-like state clearly varies between [FeFe]-hydrogenases, as *Dd*HydAB did not form a similar state under identical experimental conditions. The latter observation is tentatively attributed to *Dd*HydAB’s high affinity for CO, resulting in a relatively stable H_ox_-CO population unable to convert to the H_trans_-like state under the conditions employed here.

In light of its stability, we propose that the observed H_trans_-like state represents a thermodynamic sink for the enzyme accessed under the whole-cell conditions employed here. Moreover, its formation is evidently accelerated by mutating amino-acid residues close to the open-coordination site of the cofactor, and by the presence of an amino-group in the bridging dithiolate ligand. Models have been proposed that focus on the role of the hydrogen-bonding network in stabilizing apical ligands, therefore, it can be hypothesized that the nature of the H-bond partners in the vicinity of the apical site can significantly influence the formation of an inhibited state. [54] In addition, a cysteine-to-serine mutation has the effect of reducing the steric hindrance close to the open-coordination site of the cofactor, which could further facilitate the insertion of an inhibitor ligand. The positive effect of adding sulfide for generating this species and stabilizing it, as during isolation of [2Fe]^adt^-*Cr*HydA1-C169S, suggests that the observed rhombic EPR signal can be attributed to the in vitro characterized H_trans_ state. The observation of H_trans_ under whole-cell conditions would support a physiological relevance of this state. However, we note that the in vivo data does not unequivocally prove the structure of this species, and further investigations on the nature of this H_trans_-like state are underway.

Albeit the H_trans_-like state appears stable, it does not seem to offer substantial protection against oxygen damage, in contrast to in vitro observations [33, 34]. Conversely, *E. coli* cells were found to provide a surprisingly high level of protection versus air, highlighting the potential of *E. coli* as a suitable host organism for oxygen-sensitive enzymes in bio (hybrid) technological applications.

In closing, the spontaneous formation of inactive species could pose a problem in the development of systems that take advantage of bacterial hosts to develop whole-cell hydrogen production systems, as it potentially shortens their service life. Having discovered the critical role of the steric occupancy and the hydrogen-bonding network of the active-site pocket in regulating the kinetics of this phenomenon, a possible path for research could be to fine-tune these parameters and thus develop potentially more robust catalysts.

## Experimental procedures

**General.** All chemicals were purchased from Sigma-Aldrich or VWR and used as received unless otherwise stated. All anaerobic work was performed in an MBRAUN glovebox ([O_2_] < 10 ppm). The expression vector encoding the *hydA1* gene (pETDuet-*Cr*HydA1-His) was kindly provided by Prof. Marc Fontecave (College de France, Paris/CEA, Grenoble). (Et_4_N)_2_[Fe_2_(adt)(CO)_4_(CN)_2_] ([2Fe]^adt^]) and (Et_4_N)_2_[Fe_2_(pdt)(CO)_4_(CN)_2_] ([2Fe]^pdt^]) were synthesized in accordance to literature protocols with minor modifications, and verified by FTIR spectroscopy [55–58]. The complexes were dissolved in anaerobic potassium phosphate buffer (100 mM, pH 6.8) at 10 µg/µL concentration and used directly. Protein content was analyzed by 10% SDS-PAGE minigels in a BioRad Mini-PROTEAN Tetra Cell system. Protein bands were stained with Page Blue protein staining solution (Thermo Fisher Scientific) according to the supplier`s instructions.

**Generation of *Cr*HydA1-C169S mutant.** To generate the C169S mutant in HydA1 protein, the triplet coding for the cysteine amino acid in position 169 (TGC) was replaced with one coding for serine (TCC). The pETDuet-*Cr*HydA1-His was mutated with the Quick Change Site-Directed Mutagenesis Kit (Agilent) according to the manufacturer instructions using 5` GTTTACCAGCTCCTGCCCGGGCTGGATTGC` 3` and 5` GCAATCCAGCCCGGGCAGGAGCTGGTAAAC` 3` primers. The amino-acid change was verified by sequencing.

**Overexpression of the apo-*Cr*HydA1 hydrogenase.** *Escherichia coli* BL21(DE3) cells containing the *Cr*HydA1 plasmid were grown in 50 mL M9 medium [22 mM Na_2_HPO_4_, 22 mM KH_2_PO_4,_ 85 mM NaCl, 18 mM NH_4_Cl, 0.2 mM MgSO_4,_ 0.1 mM CaCl_2_, 0.4% (v/v) glucose] under aerobic conditions until O.D._600_ = 0.6 – 0.8 in the presence of ampicillin. The protein overproduction was induced with 1 mM IPTG and persisted at 20 °C for 16 – 18 h with continuous aeration. The media was supplemented with 100 µM FeSO_4_ at the time of the induction. Final O.D._600_ of the cultures were 1.4 ± 0.2.

**Overexpression of the apo-*Dd*HydAB hydrogenase.** *Escherichia coli* BL21(DE3) cells containing the pACYCDuet-*Dd*HydAB plasmid[44] were grown in 50 mL M9 medium [22 mM Na_2_HPO_4_, 22 mM KH_2_PO_4,_ 85 mM NaCl, 18 mM NH_4_Cl, 0.2 mM MgSO_4,_ 0.1 mM CaCl_2_], supplemented with 0.4% (w/v) glucose, under aerobic conditions until O.D._600_ = 0.6 – 0.8 in the presence of chloramphenicol. The protein overproduction was induced with 1 mM IPTG and persisted at 20 °C for 16–18 h with continuous aeration. The media was supplemented with 400 µM FeSO_4_ at the time of the induction. Final O.D._600_ of the cultures were 1.4 ± 0.2.

**In vivo formation of [2Fe]**^**adt**^***-Cr*HydA1, [2Fe]**^**pdt**^***-Cr*HydA1, [2Fe]**^**adt**^***–Dd*HydAB and [2Fe]**^**pdt**^***-Dd*HydAB.** The preparation of the semi-synthetic hydrogenase was performed following literature protocols with minor modifications [38, 39]. The apo-protein was expressed in 50 mL *E. coli* cultures as described in the “*Overexpression of the apo-CrHydA1 hydrogenase”* and “*Overexpression of the apo-DdHydAB hydrogenase”* sections. After the 16–18 h expression period the cells were harvested, deaerated and transferred to the glovebox. The cells were re-suspended in fresh M9 medium (2 mL final volume), and formation of [2Fe]^adt^–*Cr*HydA1, [2Fe]^pdt^–*Cr*HydA1, [2Fe]^adt^–*Dd*HydAB and [2Fe]^pdt^-*Dd*HydAB was achieved by incubating the cell suspensions with 100 µg (156 nmol) [2Fe]^adt^ or 100 µg (156 nmol) [2Fe]^pdt^ complex (80 μM final conc.), for 1 – 96 h at 37 ˚C under strictly anaerobic conditions. When indicated, the medium was supplemented with 375 µM L-cysteine or 375 µM Na_2_S.

For the medium exchange experiments, after 23 h incubation the cell suspensions were transferred in the glovebox, centrifuged and the resulting pellet was re-suspended in fresh M9 media, supplemented with 0.4% (w/v) glucose when necessary. The cell suspensions were then incubated for 2 h at 37 ˚C under strictly anaerobic conditions.

**Whole-cell EPR sample preparation.** The 2 mL concentrated cell suspensions generated via the “In vivo* formation of [2Fe]*^*adt*^*-HydA1, [2Fe]*^*pdt*^*-HydA1, [2Fe]*^*adt*^*–DdHydAB and [2Fe]*^*pdt*^*-DdHydAB*” protocol were centrifuged and the cell pellet washed with 1 mL TRIS–HCl buffer (100 mM TRIS, 150 mM NaCl pH 7.5) three times under anaerobic conditions. After washing, the cells were diluted to a final volume of 400 µL using the same buffer and transferred into EPR tubes. The tubes were capped and directly frozen in liquid nitrogen.

**Isolation of [2Fe]**^**adt**^***-Cr*HydA1-C169S**. 100 mL of C169S-*Cr*HydA1 cells were activated with 100 µg [2Fe]^adt^ for 1 h. After activation the cells were lysed in lysis buffer (50 mM sodium phosphate buffer pH 7.8, 100 mM NaCl, 20 µg/mL DNaseI, 40 µg/mL RNaseI, 1 mM MgCl_2_, 0.1 mg/mL lysozyme) using three freeze–thaw cycles. The soluble fraction of the cell lysate was separated with 13,000 rpm centrifugation for 20 min under anaerobic conditions. The protein was purified using a 1 mL Ni-sepharose High Performance (GE Heathcare) gravity column equilibrated with 10 column volumes of equilibration buffer (50 mM sodium phosphate buffer pH 7.8, 100 mM NaCl) in the glovebox. The non-specifically bound proteins were removed with wash buffer (50 mM sodium phosphate buffer pH 7.8, 100 mM NaCl, 20 mM imidazole). The [2Fe]-C169S-*Cr*HydA1 protein was eluted with 1.5 mL elution buffer (50 mM sodium phosphate buffer pH 7.8, 100 mM NaCl, 300 mM imidazole), the elution fraction was directly used for EPR samples. The purity of the purified protein was confirmed with SDS-PAGE.

**Hydrogenase activity measurements.** Activity measurements were performed on whole-cell samples as well as under in vitro conditions using published protocols [39]. Selected technical details are re-iterated for clarity. Whole-cell activities were determined from cell suspensions (2 mL final volume), incubated in gastight vials with a total volume of 9 mL. In vitro assays were performed on cell lysates. The reaction mix contained potassium phospate buffer (pH 6.8, 100 mM), Triton-X 1.5% v/v and methyl viologen (14 mM);the reaction was initiated with the addition of dithionite (200 mM) and the sample vials incubated for 15 min at 37 °C prior to headspace sampling. Hydrogen production was determined by analyzing the headspace gas, using a gas chromatograph (GC; PerkinElmer LLC, MA, USA) equipped with a thermal conductivity detector (TCD) and a stainless-steel column packed with Molecular Sieve (60/80 mesh). A calibration curve was established by injecting known amounts of hydrogen. The operational temperatures of the injection port, the oven and the detector were 100 °C, 80 °C and 100 °C, respectively. Argon was used as the carrier gas at a flow rate of 35 mL min^−1^.

**EPR measurements.** The EPR spectra shown are representative signals from at least two individual experiments. The individual experiments show some preparation dependent differences, but the amplitude of these background signals are negligible compared to the signal intensity of the [2Fe]^adt^ activated *Cr*HydA1. Measurements were performed on a Bruker ELEXYS E500 spectrometer using an ER049X SuperX microwave bridge in a Bruker SHQ0601 cavity equipped with an Oxford Instruments continuous flow cryostat and using an ITC 503 temperature controller (Oxford Instruments). Measurement temperatures ranged from 10 to 20 K, using liquid helium as coolant, with the following EPR settings unless otherwise stated: microwave power 1 mW, modulation amplitude 1 mT, modulation frequency 100 kHz. The spectrometer was controlled by the Xepr software package (Bruker).

**EPR spectra processing and simulations.** The EPR spectra were processed using the softwares Matlab (Mathworks, Inc) and QSoas [59]. Matlab served for converting the EPR files to ascii format, while QSoas was used to display the spectra as a function of g values, for visual inspection and subtraction of background signals emerging from the cells. The processed signals were used for Figs. 2, 3, 4, 5, 6, S2 and S3, S5 to S8. The simulations were performed using the Easyspin toolbox (5.2.23) within Matlab [60]. Other details of the procedure can be found in [38].

## Supplementary Information

Below is the link to the electronic supplementary material.

Supplementary file1 (PDF 843 KB)
