# Supplementary material for: Stability of the H-cluster under whole-cell conditions—formation of an Htrans-like state and its reactivity towards oxygen
Source: J Biol Inorg Chem. 2022 Mar 8;27(3):345–55. doi: 10.1007/s00775-022-01928-5 (PMC8960641; doi:10.1007/s00775-022-01928-5)
Supplement: Supplementary file 1 — Supplementary file1 (PDF 843 KB) [file 775_2022_1928_MOESM1_ESM.pdf]

Supplementary information

Stability of the H-cluster under whole-cell conditions – Formation of an H_{trans}-like state and its reactivity towards oxygen

Marco Lorenzi[1], Pierre Ceccaldi[1], Patricia Rodríguez-Maciá[2‡], Holly J. Redman[1], Afridi Zamader[1,3], James A. Birrell[2], Livia S. Mészáros[1*], Gustav Berggren[1*]

[1]Molecular Biomimetics, Department of Chemistry – Ångström Laboratory, Uppsala University, Box 523, SE-75120, Uppsala, Sweden. [†]Current address: R&I consultant, home office, Marseille, France.

[2]Max Planck Institute for Chemical Energy Conversion, Department of Inorganic Spectroscopy, Stiftstrasse 34-36, 45470 Mülheim an der Ruhr, Germany. [‡]Current address: Department of Chemistry, University of Oxford, Inorganic Chemistry Laboratory, South Parks Road, OX1 3QR, United-Kingdom

[3]Laboratoire de Chimie et Biologie des Métaux, Université Grenoble Alpes, CNRS, CEA, 17 rue des Martyrs, 38054 Grenoble, France

Table of Contents

Figure S1. Cumulative H_2 production from *in vivo* matured $[2Fe]^{adt}$-*Cr*HydA1 samples. 2

Figure S2. EPR spectra of cells containing $[2Fe]^{adt}$-*Cr*HydA1 incubated for 23 h and subsequently re-suspended in fresh media. 3

Figure S3. *In vitro* H_2 production activity assays performed on lysates of cells expressing apo-*Cr*HydA1 4

Figure S4. EPR spectra of control samples. 5

Figure S5. Effect of the addition of L-cysteine or sodium sulfide on the intensity of the H_{trans}-like signal in whole-cell $[2Fe]^{adt}$-*Cr*HydA1 samples.. 6

Figure S6. The effect of time on $[2Fe]^{pdt}$-CrHydA1 under whole-cell conditions monitored by EPR spectroscopy. 7

Figure S7. Isolation of [2Fe]-*Cr*HydA1-C169S. 8

Figure S8. Monitoring H-cluster assembly in whole-cells expressing *Cr*HydA1-C169S at 12 °C. 9

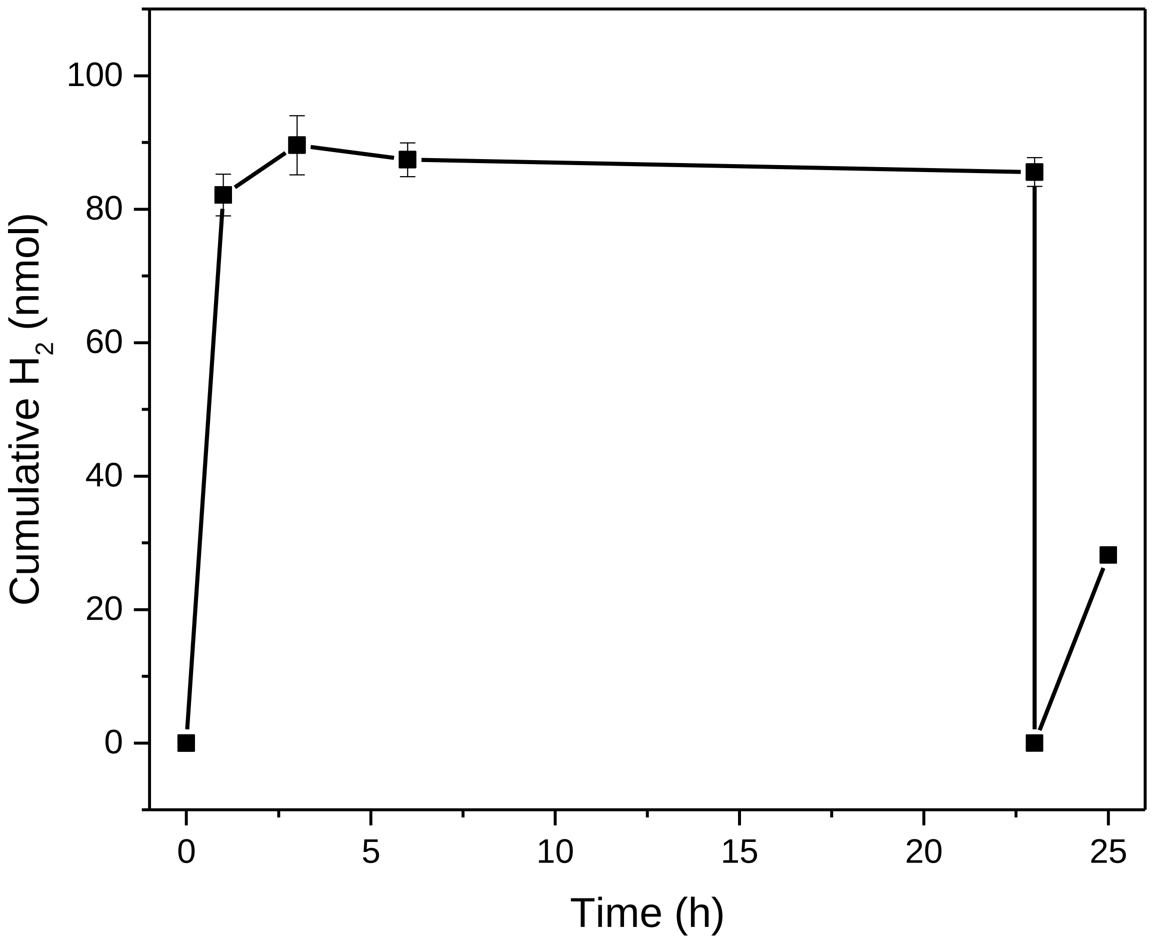

Figure S1. Cumulative H_2 production from *in vivo* matured $[2Fe]^{adt}$-*Cr*HydA1 samples.

100 mL cultures of apo-*Cr*HydA1 expressing *E. coli* cells were harvested, transferred in a glove-box, resuspended in M9 medium supplemented with 0.4% (w/v) glucose and incubated with 80 µM $[2Fe]^{adt}$ complex for 23 h at 37 °C inside sealed glass vials. Over the course of the incubation, the headspace gas of the vials was sampled and analyzed using a gas-chromatograph to monitor H_2 production. After 23 h of incubation, the cell suspensions were transferred back to the glove-box, opened to the argon atmosphere and centrifuged to replace the old medium with fresh one. They were then again incubated at 37 °C inside sealed glass vials, and the headspace gas was sampled after 2 h to verify the recovery of H_2 production.

Data points represent the average value from two biological replicates, and error bars represent the standard deviation for the two measurements.

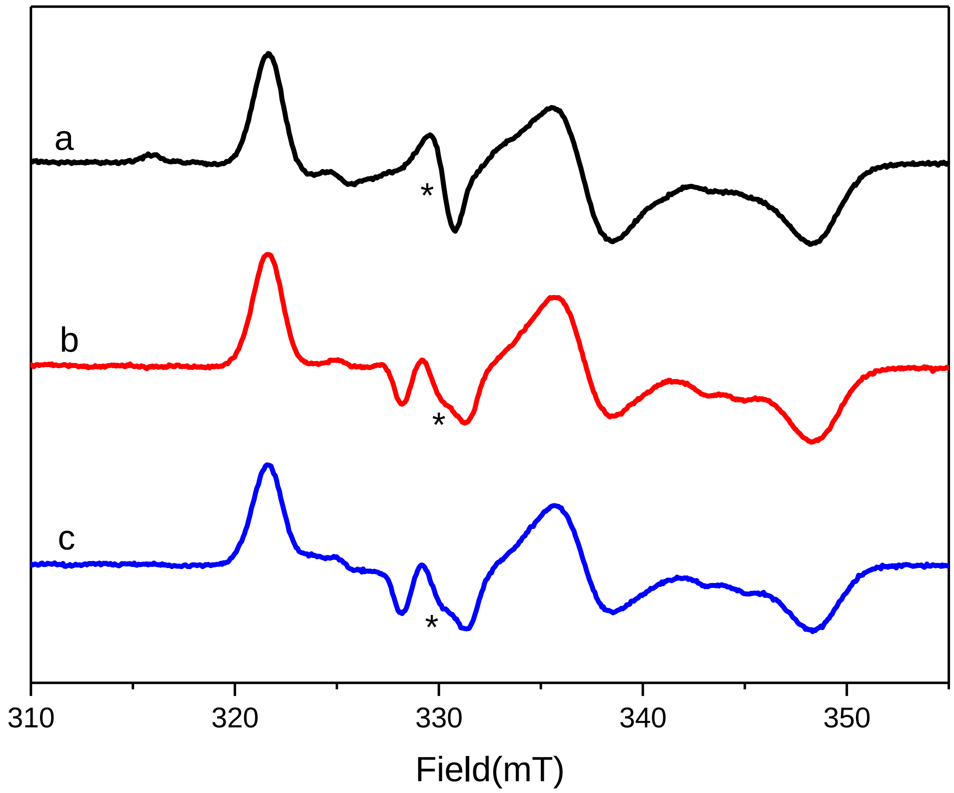

Figure S2. EPR spectra of cells containing [2Fe]^{adt}-*Cr*HydA1 incubated for 23 h and subsequently re-suspended in fresh media. (a) EPR spectrum of *apo*-*Cr*HydA1-expressing *E. coli* cells flash frozen after 25 h of incubation at 37 °C with [2Fe]^{adt} **(b)** EPR spectrum *of apo*-*Cr*HydA1-expressing *E. coli* cells flash frozen after 23 h of incubation at 37 °C with [2Fe]^{adt}, followed by 2 h additional incubation in fresh M9 media. **(c)** EPR spectrum of *apo*-*Cr*HydA1-expressing *E. coli* cells flash frozen after 23 h of incubation at 37 °C with [2Fe]^{adt} followed by 2 h additional incubation in fresh M9 media complemented with 0.4% glucose. All three spectra display a predominance of H_{trans}-like features with comparable intensities. EPR experimental conditions: T = 10 K, P = 1 mW, ν = 9.28 GHz.

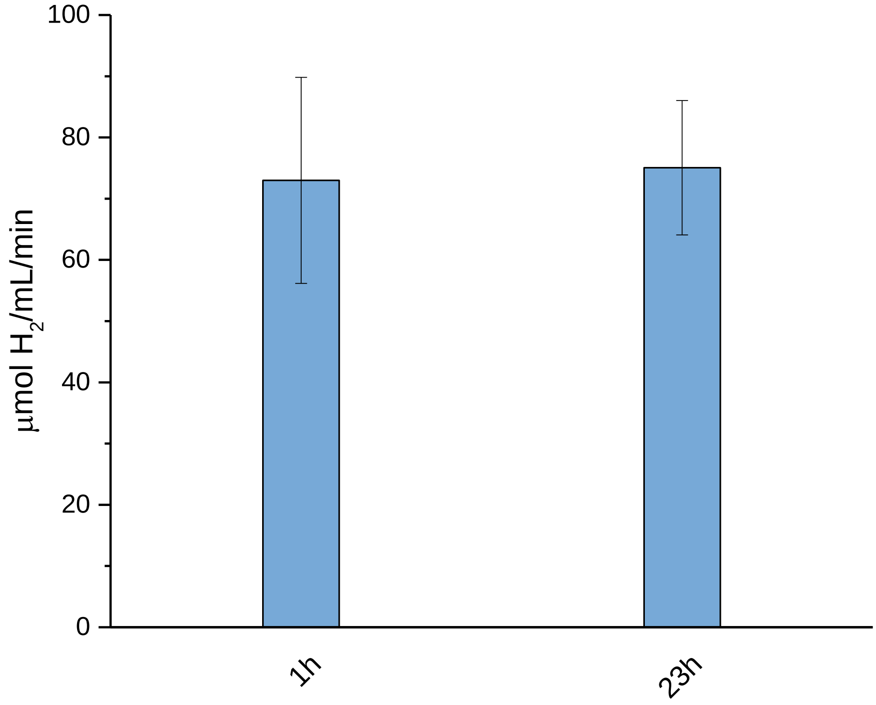

Figure S3. *In vitro* H_2 production activity assays performed on lysates of cells expressing apo-*Cr*HydA1. 10 mL of a culture of *E. coli* cells expressing apo-*Cr*HydA1 were harvested, transferred inside a glovebox, resuspended in M9 medium supplemented with 0.4% (w/v) glucose and incubated for 1 or 23 h at 37 °C, inside sealed glass vials. Then, the cell suspensions where centrifuged and the cells were anaerobically lysed using a lysis buffer. 15.6 nmol of $[2Fe]^{adt}$ was then added to the lysates before transferring them into clean vials together with 1.42 mL of assay buffer (84.5 mM potassium phospate buffer, pH 6.8, 1.4% Triton X-100 and 14 mM methyl viologen). The vials were sealed and transferred to a 37 °C bath, and H_2 production was started injecting 200 µl of 0.2 M sodium dithionite. Headspace gas was sampled and analyzed using a gas chromatograph 15 minutes after the reaction started.

As no significant difference in activity between samples incubated for 1 h and for 23 h was observed, the amount of *apo*-*Cr*HydA1 available for artificial maturation was identical within error. This indicates that protein degradation is negligible on the timescale considered here. Error bars represent the standard deviation on average activity from 4 biological replicates.

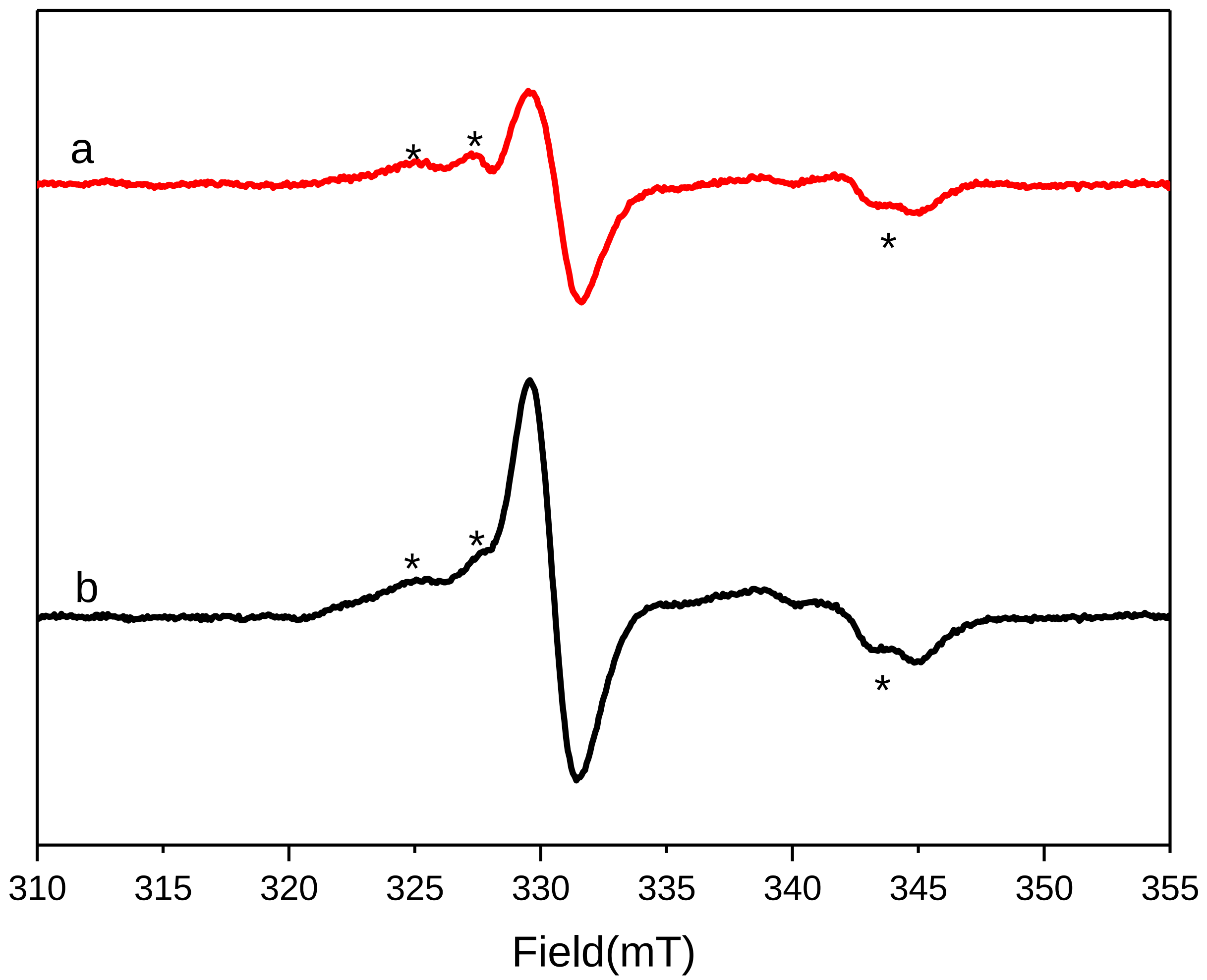

Figure S4. EPR spectra of control samples. (a) EPR spectrum of *E. coli* cells expressing *apo-Cr*HydA1 flash frozen after 23 h incubation under anaerobic conditions at 37 ˚C in the absence of $[2Fe]^{adt}$. **(b)** EPR spectrum of BL21 cells flash frozen after 23 h incubation with $[2Fe]^{adt}$ under anaerobic conditions at 37 ˚C. In both spectra, the dominant feature is the almost isotropic signal around 330 mT (g = 2.02), additional (minor) signals marked with asterisks are attributable to a $[4Fe\text{-}4S]^{+}$ species. Both these features are attributable to intrinsic *E. coli* paramagnetic species and represent a variable and sample-dependent contribution to every whole-cell EPR spectrum; therefore, subtracting these backgrounds from whole-cell EPR spectra results in spectra displaying a residual feature around 330 mT or g = 2.02. EPR experimental conditions: T = 20 K, P = 1 mW, ν = 9.28 GHz, the cavity signal has been subtracted for clarity.

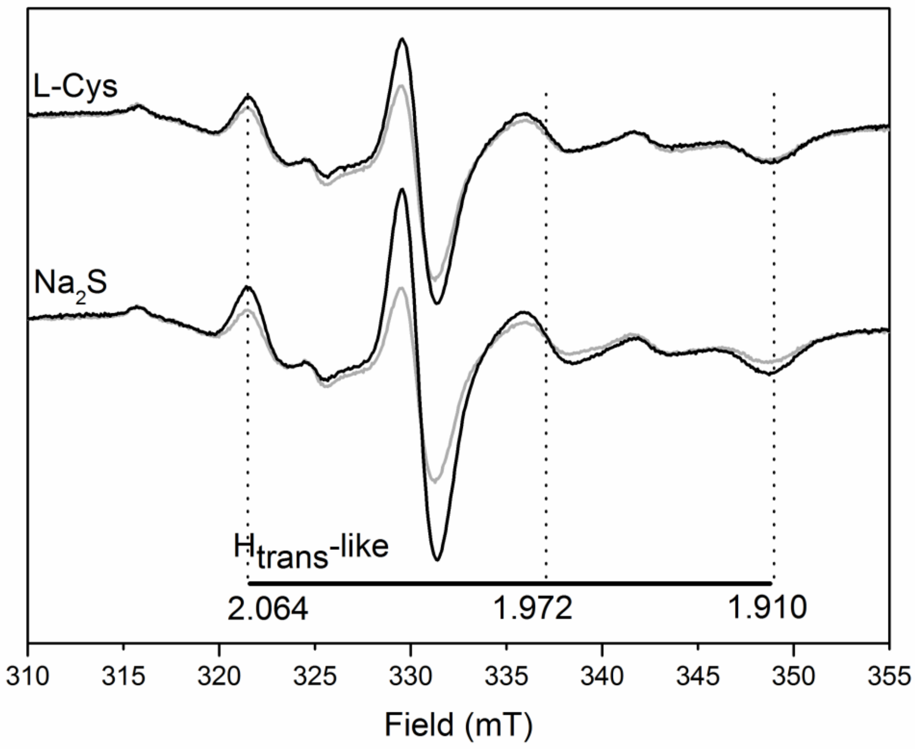

Figure S5. Effect of the addition of L-cysteine or sodium sulfide on the intensity of the H_{trans}-like signal in whole-cell $[2Fe]^{adt}$-*Cr*HydA1 samples. EPR spectra of *apo*-*Cr*HydA1-expressing *E. coli* cells flash frozen after 23 h of incubation at 37 °C with 80 µM $[2Fe]^{adt}$ in the presence of 375 µM L-cysteine (**L-Cys, upper black trace**) or 375 µM sodium sulfide (**Na_2S, lower black trace**). Both spectra are overlaid with the spectrum of a control sample prepared under the same conditions but without addition of cysteine or Na_2S (**gray traces**). Addition of Na_2S increases the intensity of the spectral features attributed to the H_{trans}-like state by approx. 50%. A weaker but still discernable increase is observed also with L-cysteine. EPR experimental conditions: T = 20 K, P = 1 mW, ν = 9.28 GHz.

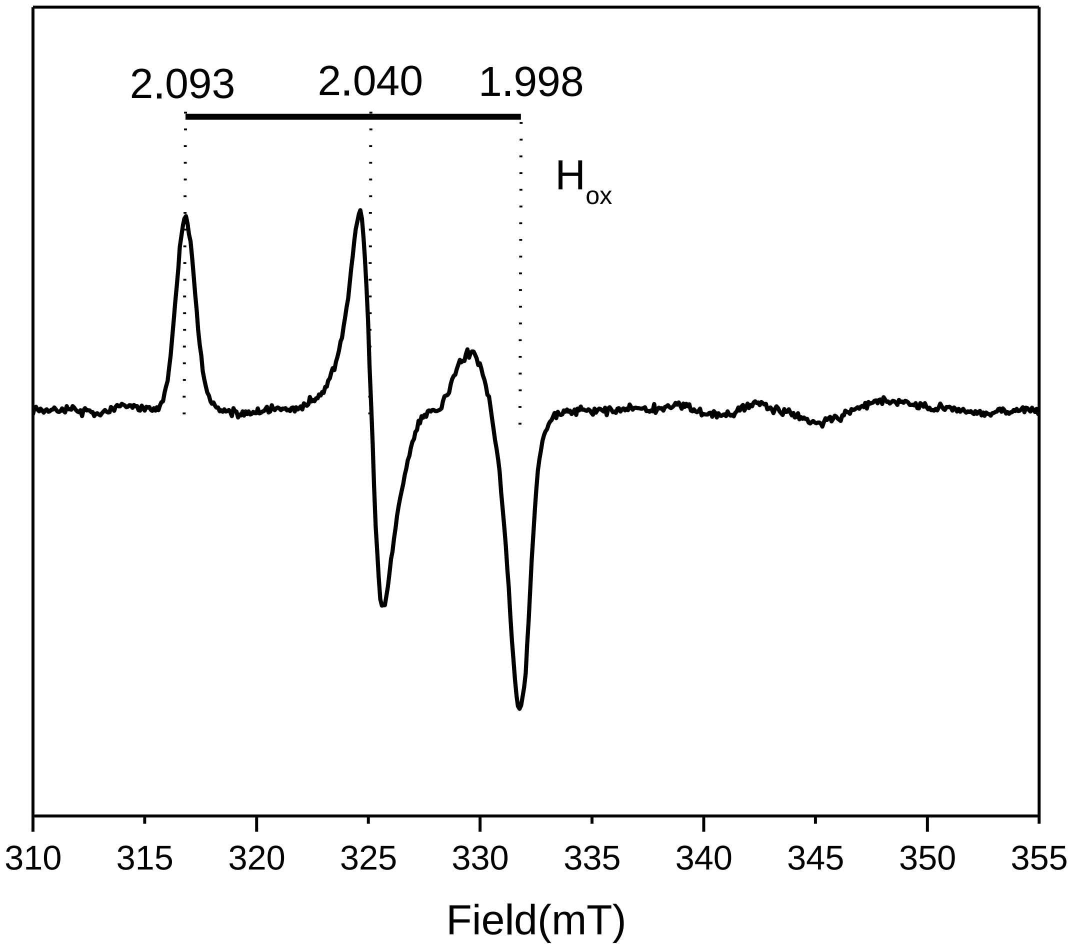

Figure S6. The effect of time on $[2Fe]^{pdt}$-CrHydA1 under whole-cell conditions monitored by EPR spectroscopy. EPR spectra of *apo*-*Cr*HydA1-expressing *E.coli* cells frozen after 23 h of incubation at 37 °C with 80 μM $[2Fe]^{pdt}$. Despite the long incubation, the sample displayed a typical H_{ox} signature and did not show any feature attributable to an H_{trans}-like state. EPR experimental conditions: T = 10 K, P = 1 mW, ν = 9.28 GHz.

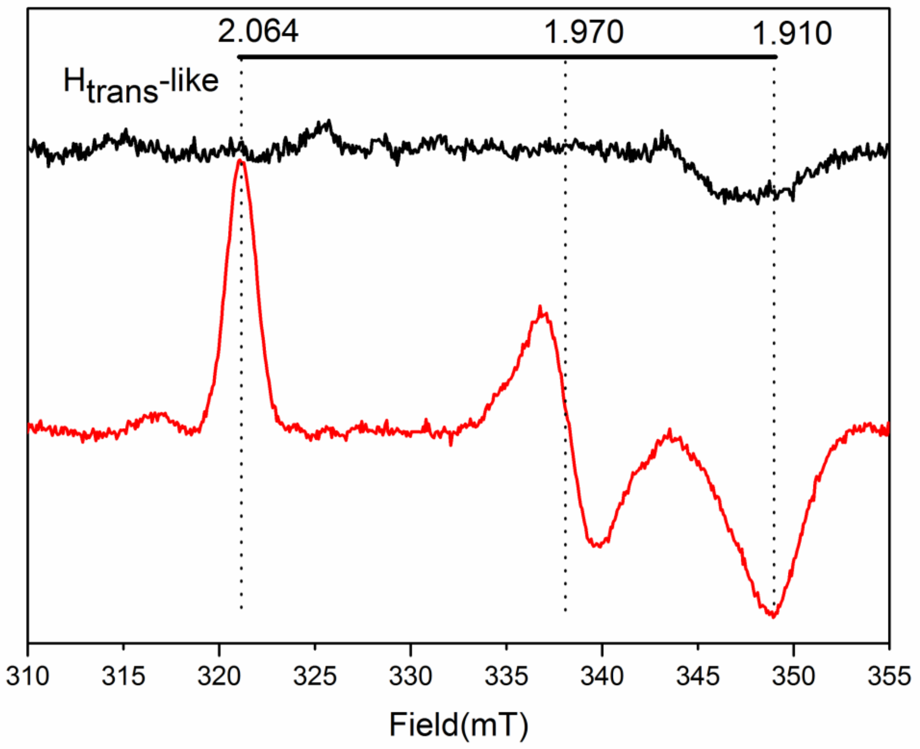

Figure S7. Isolation of [2Fe]-*Cr*HydA1-C169S. EPR spectra recorded of purified samples of $[2Fe]^{adt}$-HydA1-C169S. The samples were isolated under strictly anaerobic conditions via affinity chromatography from cell lysates of *apo*-*Cr*HydA1-C169S expressing *E.coli* cells lysed after 1 h of incubation at 37 °C with 80 μM $[2Fe]^{adt}$. The $[2Fe]^{adt}$-HydA1-C169S protein was isolated in buffer lacking Na_2S **(black spectrum)**; or buffer supplemented with 100 mM Na_2S **(red spectrum)**. Features attributed to the H_{trans}-like state of $[2Fe]^{adt}$-HydA1-C169S (g_{zyx}= 2.064, 1.970, 1.910) were readily observable in samples isolated using buffers supplemented with 100 mM Na_2S. EPR experimental conditions: T = 10 K, P = 1 mW, ν = 9.28 GHz.

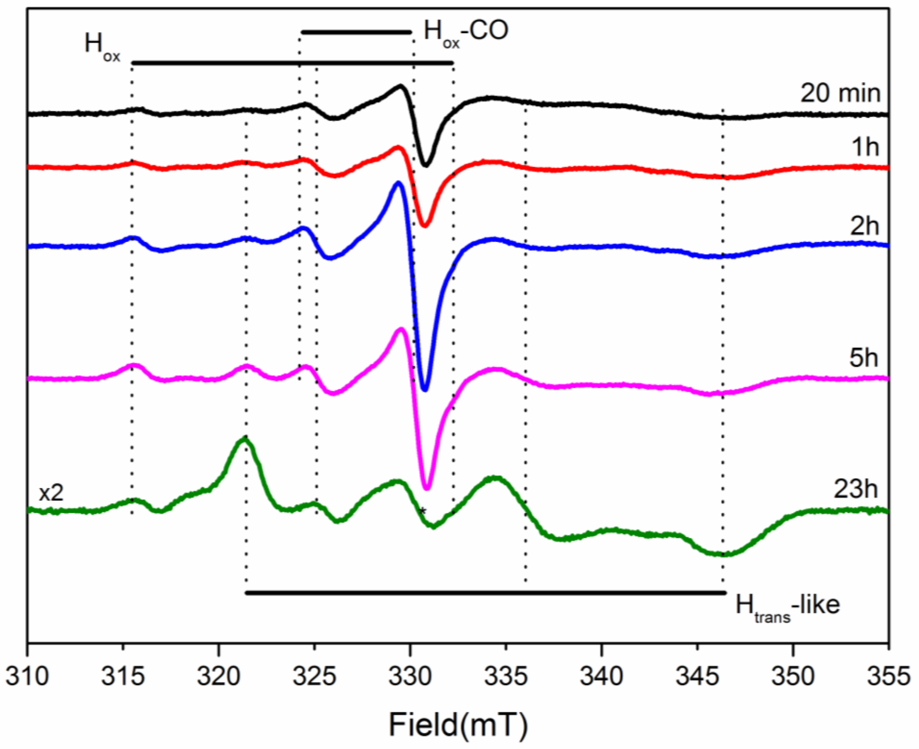

Figure S8. Monitoring H-cluster assembly in whole-cells expressing *Cr*HydA1-C169S at 12 ºC. EPR spectra recorded of apo-HydA1-C169S expressing *E. coli* cells incubated with $[2Fe]^{adt}$ at 12 ˚C, and flash frozen after 20 minutes **(black spectrum)**; 1 h **(red spectrum)**; 2 h **(blue spectrum)**; 5 h **(purple spectrum)**; 23 h **(green spectrum)**. Signals assigned to the H_{ox}; H_{ox}-CO and H_{trans}-like state indicated with black solid horizontal lines; individual g-values are indicated with dashed vertical lines. Low temperature activation enabled the detection of intermediate H-cluster states formed prior to the H_{trans}-like state. From 20 minutes to 5 h a mixture of H_{ox} and H_{ox}-CO states are clearly discernable. Features attributed to the H_{trans}-state (g_{xyz}= 2.064, 1.970, 1.910) were present already in the 20 minute spectrum, but became the dominant species only after 23 h incubation at 12 ºC. EPR experimental conditions: T = 10 K, P = 1 mW, ν = 9.28 GHz.
